# Supplementary material for: Nuclear microRNA 9 mediates G-quadruplex formation and 3D genome organization during TGF-β-induced transcription
Source: Nat Commun. 2024 Dec 20;15:10711. doi: 10.1038/s41467-024-54740-x (PMC11662019; doi:10.1038/s41467-024-54740-x)
Supplement: Supplementary file 1 — Supplementary Information [file 41467_2024_54740_MOESM1_ESM.pdf]

SUPPLEMENTARY INFORMATION for

# Nuclear microRNA 9 mediates G-quadruplex formation and 3D genome organization during TGF-β-induced transcription

**Authors:** Julio Cordero[1, 2, 3, *], Guruprasadh Swaminathan[4], Diana G Rogel-Ayala[3, 4], Karla Rubio[3, 4, 5, 6], Adel Elsherbiny[1, 2], Samina Mahmood[7], Witold Szymanski[8], Johannes Graumann[8], Thomas Braun[9], Stefan Günther[7, 9], Gergana Dobreva[1, 2], and Guillermo Barreto[3, 4, *, †]

[†]Lead contact

* Correspondence with equal contribution to: Guillermo.Barreto@univ-lorraine.fr AND Julio.Cordero@medma.uni-heidelberg.de

**This PDF file includes:**

Supplementary Figures 1 to 9

Supplementary Tables 1 and 2

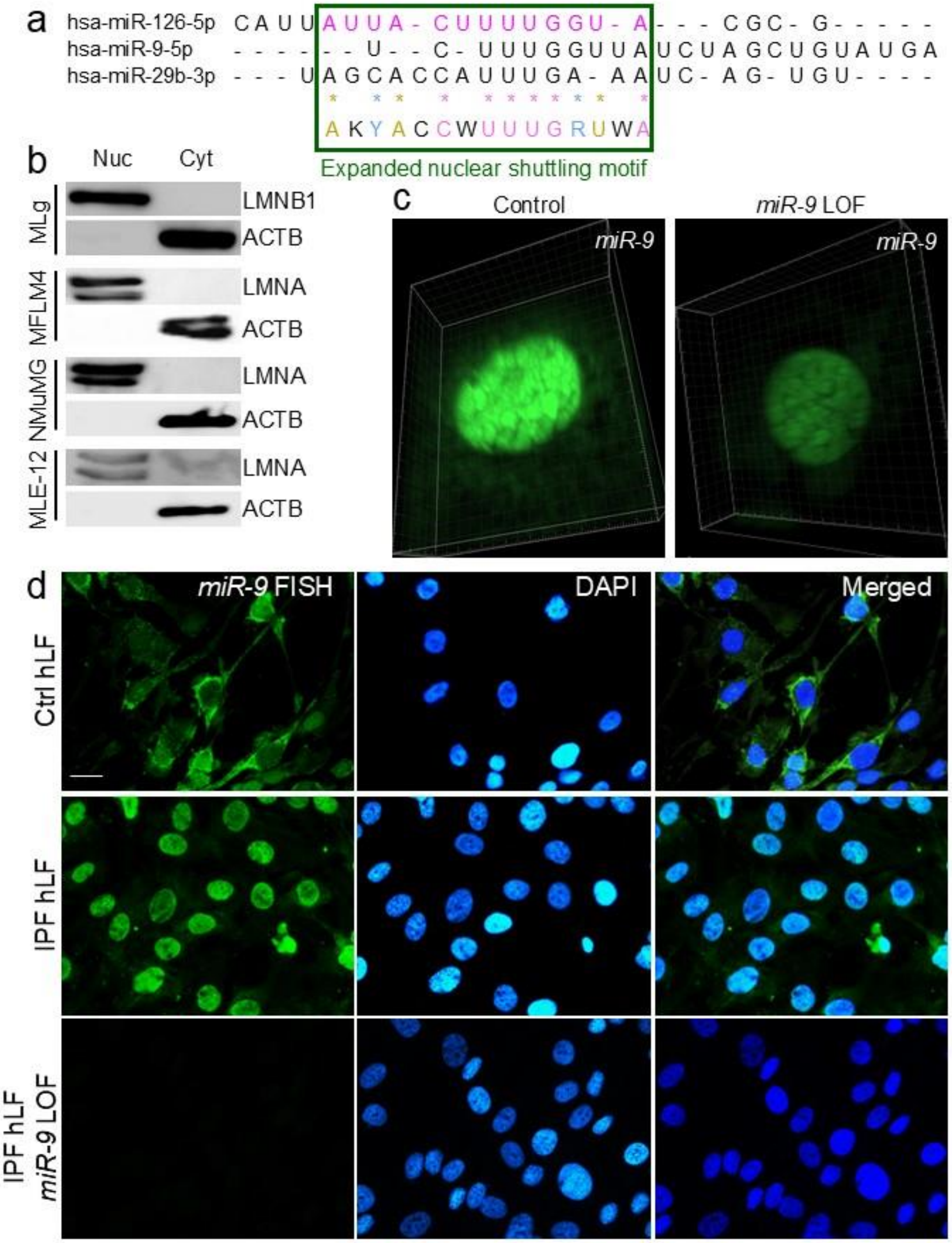


**Supplementary Figure 1: Mature *miR-9* is detected in the cell nucleus.** (**a**) Sequence alignment of the indicated mature human miRNAs highlighting the published *miR-126* nuclear shuttling motif (magenta) and the expanded miRNA nuclear shuttling motif (green square) using the IUPAC

nucleotide code. The bottom line shows the partially conserved sequence. Pink letters are conserved among all sequences. Golden letters are conserved in at least 2 sequences. Blue letters indicate conserved type of base (either purine or pyrimidine). (**b**) Cell fractionation efficiency shown by Western Blot analysis of the nuclear (Nuc) and cytosolic (Cyt) fractions of the cell lines used for TaqMan assay-based expression analysis of mature *miR-9* in Figure 1b. LMNB1, lamin B1, LMNA, lamin A/C, both nuclear markers; ACTB, beta actin, cytosolic marker. (**c**) Confocal microscopy of MLg cells after RNA FISH confirmed *miR-9* nuclear localization (left) and *miR-9* loss-of-function after *miR-9*-specific antagomiR probes transfection (right). (**d**) Fluorescence microscopy of hLF cells after RNA FISH confirmed *miR-9* cytosolic localization in control hLF (top), *miR-9* nuclear localization in IPF hLF (middle) and *miR-9* loss-of-function after *miR-9*-specific antagomiR probes transfection (bottom).

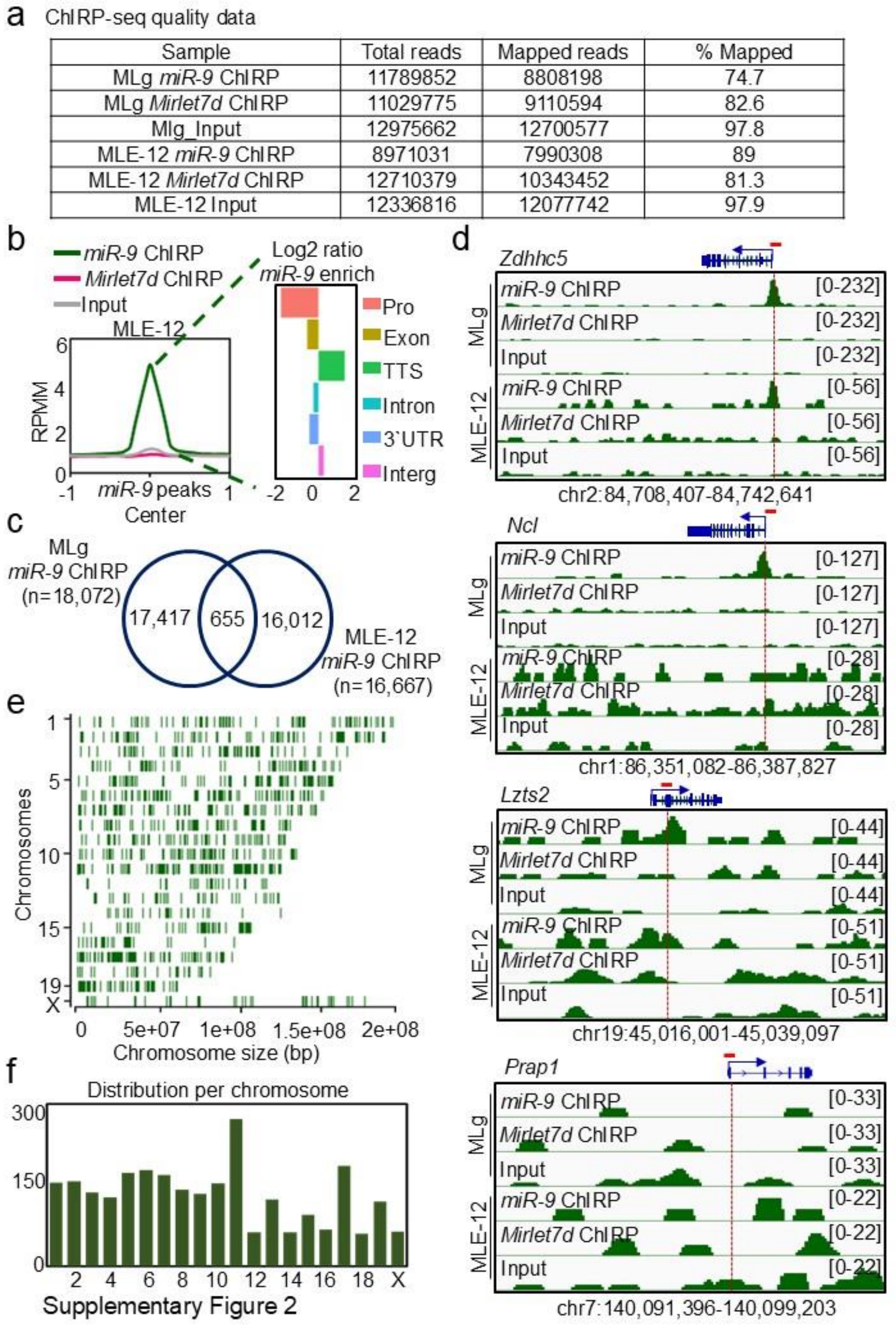


**Supplementary Figure 2: Mature *miR-9* is enriched at promoters and intronic regions and its loci are distributed on all chromosomes.** (**a**) ChIRP-seq data sets supports the quality of the

experiments. (**b**) Left, enrichment plot after *miR-9*-, or *Mirlet7d*-specific ChIRP-seq in MLE-12 cells. *Mirlet7d*-specific probe was used as negative control. RPKM, reads per kilobase of transcript per million mapped reads. Right, genome-wide distribution of *miR-9* peaks by ChIRP-seq in MLg cells in different gene structures and represented as Log2 ratios. Pro, promoters; TTS, transcription termination sites; Intron, intronic regions; 3´UTR, 3´untraslated regions; Interg, intergenic regions. (**c**) Venn diagram after cross analysis of *miR-9* ChIRP-seq in MLg and MLE-12 cells showing the common loci (n = 655), indicating cell-type specific enrichment. (**d**) Visualization of selected *miR-9* target genes using IGV genome browser showing *miR-9* or *Mirlet7d* enrichment in MLg and MLE-12 cells. ChIRP-seq reads were normalized using RPKM measure and are represented as log2 enrichment over their corresponding inputs. Images show the indicated gene loci with their genomic coordinates. Arrows, direction of the genes; blue boxes, exons; red line, regions selected for single gene analysis. (**e**) Distribution of putative *miR-9* target loci on all chromosomes. (**f**) Number of putative *miR-9* target loci in each chromosome.

a H3K4me3 C&T quality data

| Sample | Total reads | Mapped reads | % Mapped |
|---|---|---|---|
| MLg Control | 20395351 | 19926257 | 97.7 |
| MLg *miR-9*-LOF | 25376437 | 24780090 | 97.65 |
| MLE-12 Control | 27902299 | 27536778 | 98.69 |
| MLE-12 *miR-9*-LOF | 25062236 | 24776526 | 98.86 |

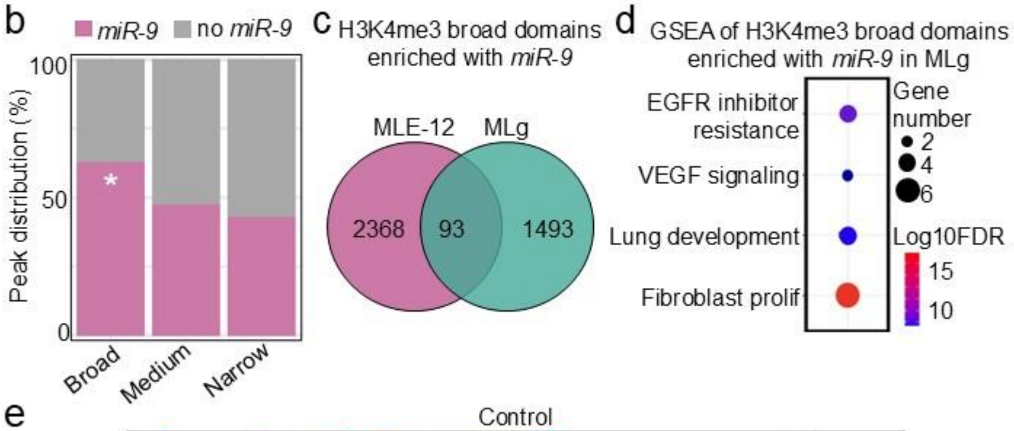


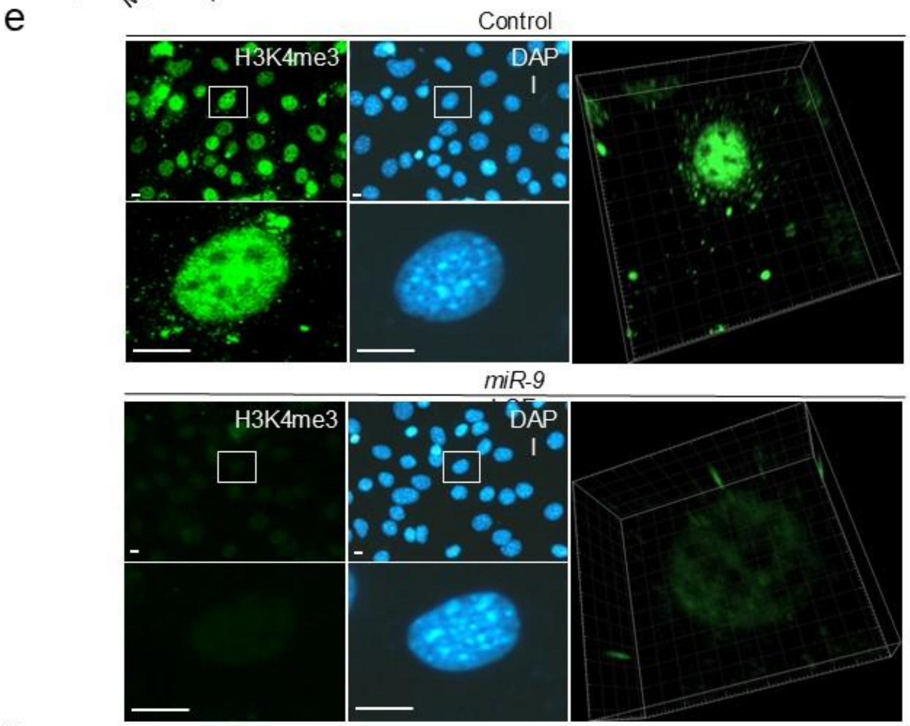


Supplementary Figure 3

f RNA seq quality data

| Sample | Total reads | Mapped reads | % Mapped |
|---|---|---|---|
| Control | 59,681,681 | 41,776,036 | 70 |
| *miR-9*-LOF | 50,055,390 | 36,778,286 | 73.48 |

**Supplementary Figure 3: *MiR-9* is required for H3K4me3 broad domains.** (**a**) Description of the CUT&Tag data set supports the quality of the experiment in MLg and MLE-12 cells. (**b**)

Genome-wide distribution of H3K4me3 peaks relative to broad ($\geq$ 2.7 kb), medium ($\geq$ 2 kb and < 2.7 kb) narrow (< 2 kb) H3K4me3 domains in MLE-12 cells that were transiently transfected with control (Ctrl) or miR-9-specific antagomiR probes to induce miR-9 loss-of-function (LOF). Asterisks, P-values after two-tailed t-test, **P $\leq$ 0.01. (c) Venn diagram of H3K4me3 broad domains decreased after miR-9 LOF in MLg and MLE-12 cells. (**d**) Gene set enrichment analysis of the H3K4me3 broad domains showing a decrease after *miR-9* LOF in MLg cells. (**e**) Fluorescence (left) and confocal (right) microscopy after H3K4me3-specific immunostaining of MLg cells transfected with control (top) or miR-9-specific (bottom) antagomiR probes to induce a miR-9 loss-of-function (LOF) confirmed the reduction of H3K4me3 broad domains. Representative images from three independent experiments. Squares are shown at higher magnification at the bottom of the respective picture. Three-dimensional confocal images from single-cells (right). DAPI, nucleus. Scale bars, 10 µm. (**f**) Description of the RNA-seq data sets supports the quality of the experiment.

a G4 CUT&Tag quality data

| Sample | Total reads | Trimmed reads | Mapped reads | % Mapped |
|---|---|---|---|---|
| MLg Control | 29177316 | 26612321 | 22567248 | 84.8 |
| MLg *miR-9-LOF* | 28889783 | 26843707 | 22567248 | 82.8 |
| MLE-12 Control | 37445818 | 34640798 | 32839476 | 94.8 |
| MLE-12 *miR-9-LOF* | 27838959 | 25810882 | 23255604 | 90.1 |

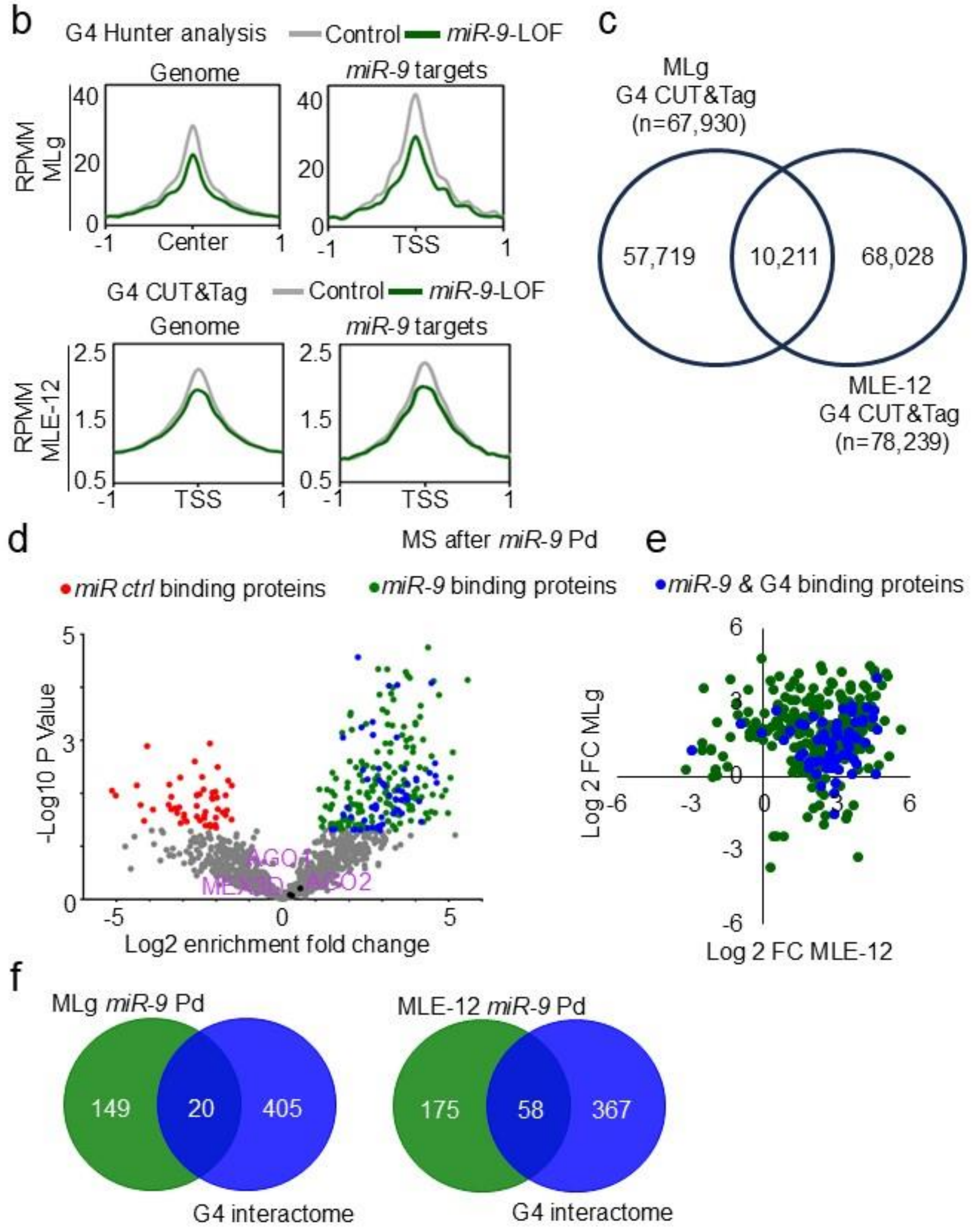


**Supplementary Figure 4: *MiR-9* is required for G-quadruplex formation and interacts with G4-reated proteins.** (**a**) G4 CUT&Tag data sets supports the quality of the experiments. (**b**)

Enrichment plots of G4-specific CUT&Tag data after filtering based on G4Hunter scores in MLg cells (top) and enrichment plots after G4-specific CUT&Tag in MLE-12 cells (bottom) that were transiently transfected with control (Ctrl) or *miR-9*-specific antagomiR probes to induce *miR-9* loss-of-function (LOF). Data were normalized using RPKM. (**c**) Venn diagram after cross analysis G4-specific CUT&Tag in MLg and MLE-12 cells showing the common loci (n = 10,211), indicating cell-type specific enrichment. (**d**) Mass spectrometry of proteins after miRNA pulldown (miR-Pd) using the nuclear fraction of MLE-12 cells and biotinylated control miRNA (*mirctrl*) or *miR-9* as baits. Volcano plot representing the significance (-log10 P-values after limma moderated t-test) vs. enrichment fold change (log2 enrichment ratios) between *miR-9*-Pd and *mirctrl*-Pd. Each dot represents a protein; green dots, proteins significantly enriched by *miR-9*; blue dots, proteins significantly enriched by *miR-9* and interacting with G4s [PMID 34188089]; red dots, proteins significantly enriched by *mirctrl*; grey and black dots, non-significantly bound proteins. Black dots show AGO1, AGOI2 and MEX3D. (**e**) Scatter plot of log2 enrichment fold change in MLE-12 (X axis) and MLg (Y axis). Each dot represents a protein; green dots, proteins significantly enriched by *miR-9*; blue dots, proteins significantly enriched by *miR-9* and interacting with G4s [PMID 34188089]. (**f**) Venn diagram after cross-analysis of *miR-9* (green) and G4 (blue) protein interactomes in MLg (left, common interacting proteins n = 20) and MLE-12 cells (right, common interacting proteins n = 58).

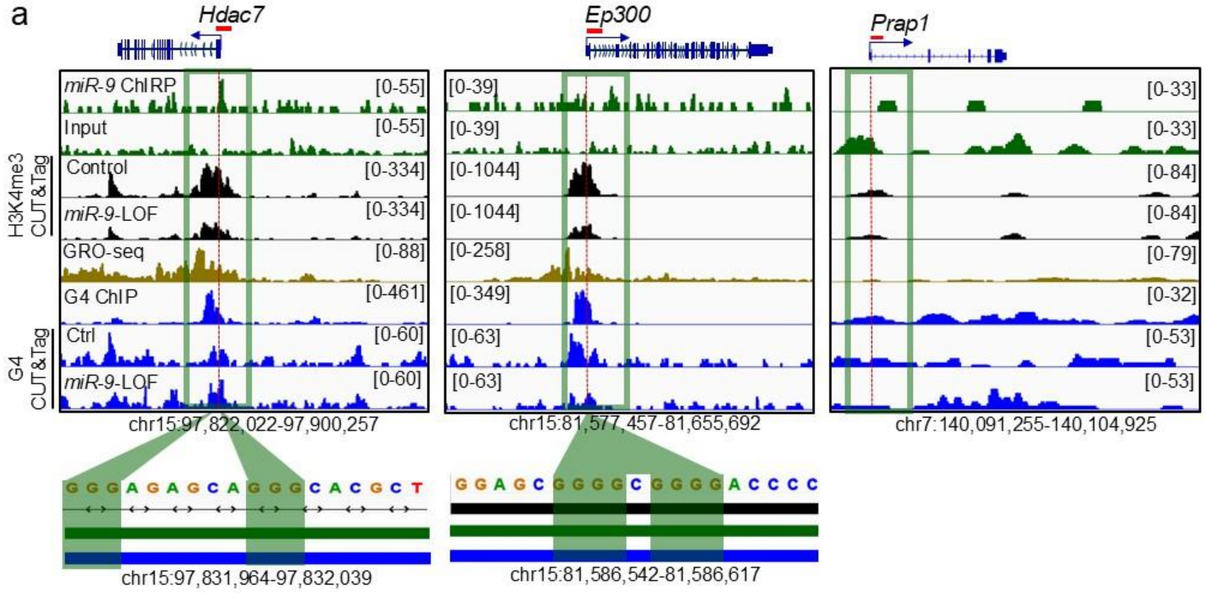


b H3K4me3 ChIP seq quality data

| Sample name | Total reads | Mapped reads | % Mapped |
|---|---|---|---|
| Control | 44,049,013 | 42,309,773 | 96.05 |
| *miR-9*-LOF | 37,138,655 | 35,556,352 | 95.74 |

**Supplementary Figure 5: H3K4me3, nascent RNA and G4 are enriched at promoters of selected *miR-9* target genes.** (**a**) Visualization of selected *miR-9* target genes (*Hdac7* and *Ep300*) using IGV genome browser showing enrichment of *miR-9* by ChIRP-seq (green), H3K4me3 by ChIP-seq in Ctrl and *miR-9*-specifc antagomiR transfected MLg cells (black), nascent RNA by GRO-seq (brown), G4 by G4P ChIP-seq in NIH/3T3 cells (blue), G4 by CUT&Tag in Ctrl and *miR-9*-specifc antagomiR transfected MLg cells (blue). Reads were normalized using reads per kilobase per million (RPKM) after bamCoverage. Images show the indicated gene loci with their genomic coordinates. Arrows, direction of the genes; blue boxes, exons; red lines, regions selected for single gene analysis; green squares, regions with enrichment of *miR-9*, H3K4me3, nascent RNA and G4; dotted lines, regions shown at the bottom with high G content. Bottom, black line,

H3K4me3 enrichment; green line, *miR-9* enrichment; blue line, G4 enrichment. (**b**) H3K4me3 ChIP-seq data sets support the quality of the experiments.

a

H3K27ac CUT&Tag quality data

| Sample | Total reads | Mapped reads | % Mapped |
|---|---|---|---|
| MLg Control | 16844878 | 16204772 | 96.2 |
| MLg *miR-9*-LOF | 17576804 | 15256665 | 86.8 |

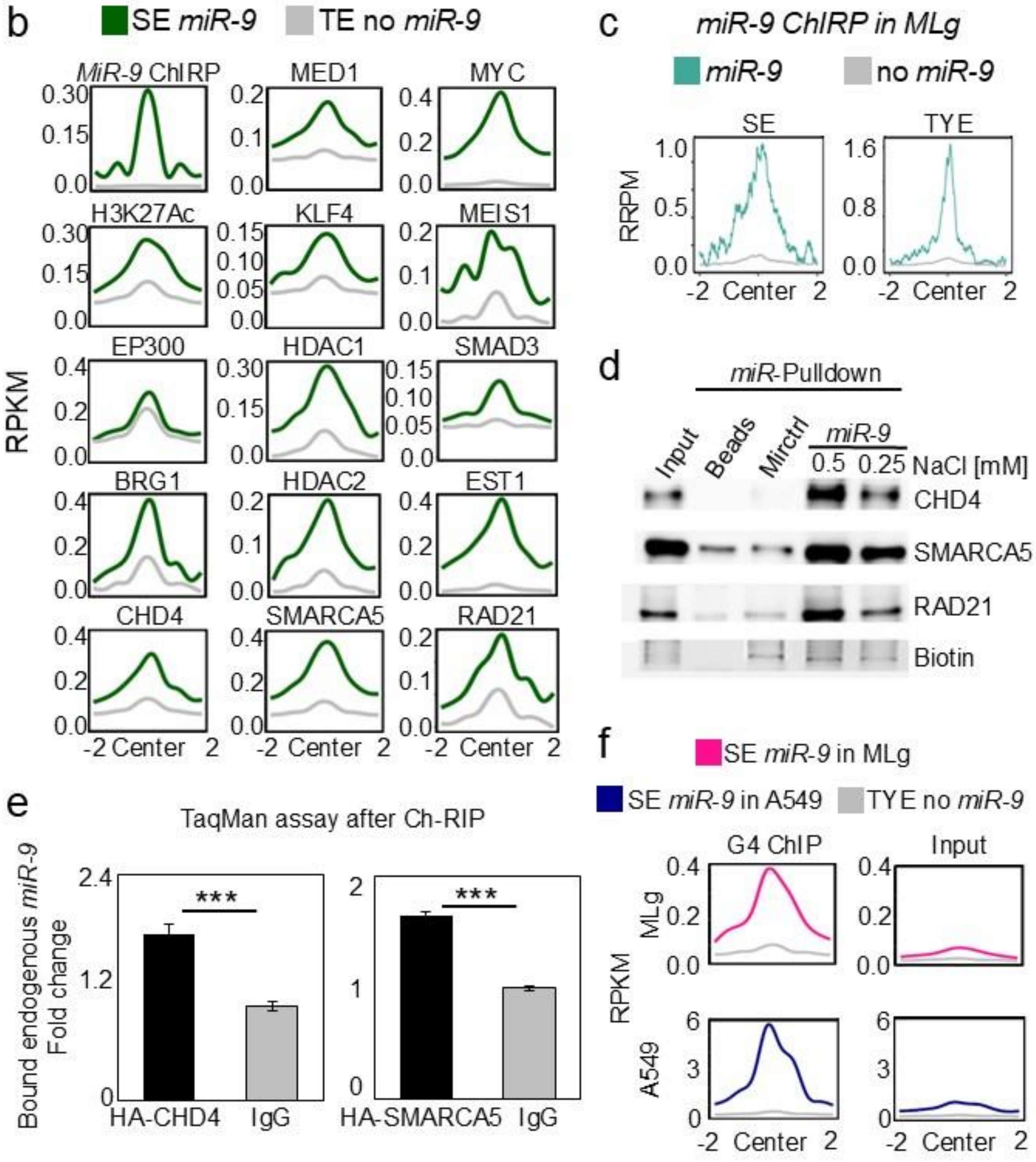


**Supplementary Figure 6: Nuclear miR-9 is enriched at super-enhancers.** (**a**) Description of the H3K27 ac CUT&Tag data set supports the quality of the experiment. (**b**) Aggregate plots

showing at SE (green lines) and TYE (grey lines) ± 2 kb the enrichment of miR-9 by ChIRP-seq in MLg and the indicated proteins by ChIP-seq, which have been previously related to SE in various murine cells. Reads were normalized using reads per kilobase per million (RPKM) measure. (**c**) Aggregate plots showing at SE (left) and TYE (right) ± 2 kb the enrichment of miR-9 by ChIRP-seq in MLE-12 (**d**)Western blot of precipitated proteins after micro RNA pulldown (*miR*-Pulldown) using the nuclear fraction from MLE-12 cells transfected with biotinylated *Mirctrl* or *miR-9*. Input, 5% of starting material. Beads, negative control without biotinylated probe. *Mirctrl*, biotinylated scramble *miRNA*. *miR-9*, biotinylated *mmu-miR-9-5p*-RNA. NaCl in milimolar (mM). (**e**) Mature *miR-9*-specific TaqMan assays after chromatin RNA immunoprecipitation (Ch-RIP) using chromatin from MLE-12 cells transfected with expression constructs of HA-CHD4 or HA-SMARCA5 fusion proteins and HA-specific antibodies or IgG (negative control). Bar plots presenting fold change as means; error bars, s.e.m ($n$ = 3 biologically independent experiments); asterisks, $P$-values after two-tailed t-test, $***P \leq 0.001$. Source data are provided as a Source Data file 01. (**f**) Aggregate plots showing at SE (magenta and blue lines) and TE (grey lines) ± 2 kb the enrichment of G4 by ChIP-seq in mouse MLg and human A549 cells. Reads were normalized using RPKM.

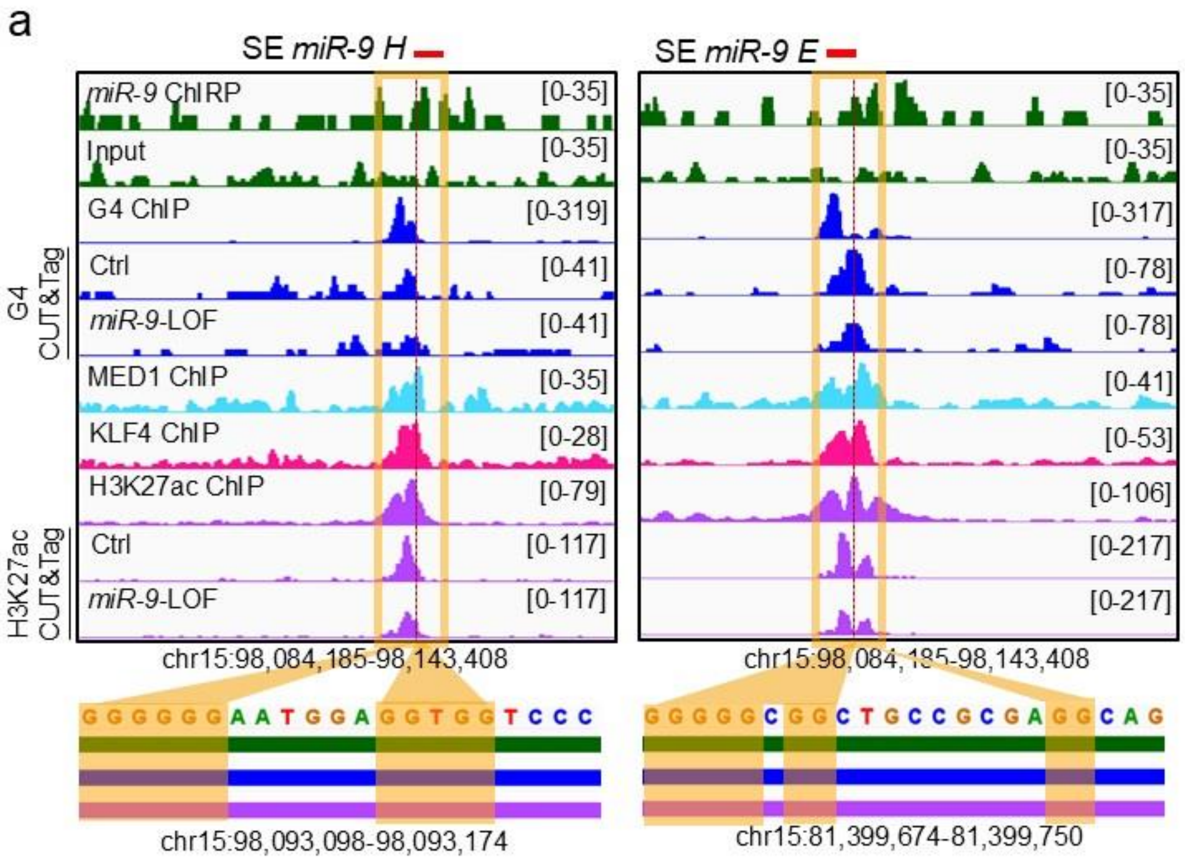


**Supplementary Figure 7: Nuclear *miR-9* is enriched at super-enhancers and is required for G-quadruplexes.** (**a**) Visualization of selected SE (*SE miR-9 H* and *SE miR-9 E*) with *miR-9* enrichment using IGV genome browser showing enrichment *miR-9* by ChIRP-seq (green), G4 by G4P ChIP-seq in NIH/3T3 cells (blue), G4 by CUT&Tag in Ctrl and *miR-9*-specifc antagomiR transfected MLg cells (blue), MED1 (turquoise), KLF4 (magenta) and H3K27ac (purple) in by ChIP-seq in mouse embryonic fibroblasts, H3K27ac by CUT&Tag in Ctrl and *miR-9*-specifc antagomiR transfected MLg cells (purple). Reads were normalized using reads per kilobase per million (RPKM). Images show the indicated gene loci with their genomic coordinates. Orange squares, regions with enrichment of *miR-9*, G4 and SE markers; red lines, regions selected for single gene analysis; dotted lines, regions shown at the bottom with high G content. Bottom, green line, *miR-9* enrichment; blue line, G4 enrichment; purple line, H3K27ac enrichment.

a H3K4me3 ChIP seq quality data

| Sample name | Total reads | Mapped reads | % Mapped |
|---|---|---|---|
| Control | 44,049,013 | 42,309,773 | 96.05 |
| TGFB1 | 34,595,383 | 33,153,192 | 95.83 |
| *miR-9*-LOF | 37,138,655 | 35,556,352 | 95.74 |

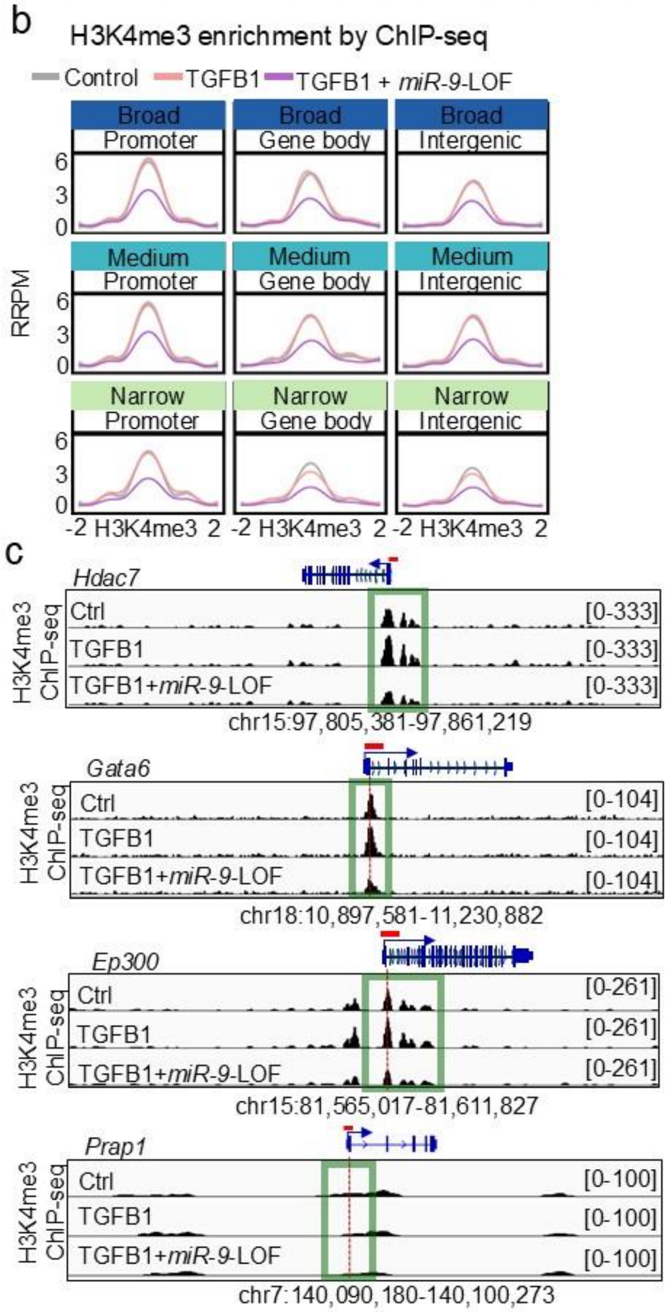


Supplementary figure 8

**Supplementary Figure 8: *MiR-9* is required for H3K4me3 enrichment in the promoter region of TGFB1-responsive genes.** (**a**) H3K4me3 ChIP-seq data sets supports the quality of the

experiments. (**b**) Aggregate plots showing H3K4me3 enrichment at the indicated gene structures and relative to broad, medium and narrow H3K4me3 domains in MLg cells in Control, TGFB1 treated and TGFB1 treated + *miR-9* LOF conditions. Data were normalized using RRPM. (**c**) Visualization of miR-9 target genes using IGV genome browser showing enrichment H3K4me3 by ChIP-seq in MLg cells that were transfected with control (Ctrl) or miR-9-specific antagomir to induce a loss-of-function (LOF), and non-treated or treated with TGFB1, as indicated. Images show the indicated loci with their genomic coordinates. Arrows, transcription direction; green squares, promoter regions; dotted lines, regions selected for single gene analysis.

**a** HiChIP-seq quality data

| Sample name | Total reads | Full reads mapped | Trimmed reads mapped | Unmaped | % Mapped |
|---|---|---|---|---|---|
| Control | 44049013 | 2840247 | 39469526 | 1739240 | 96.05 |
| TGFB1 | 34595383 | 2010415 | 31142777 | 1442191 | 95.83 |
| TGFB1+*miR9*-LOF | 44630964 | 2504104 | 40316495 | 1810365 | 95.94 |

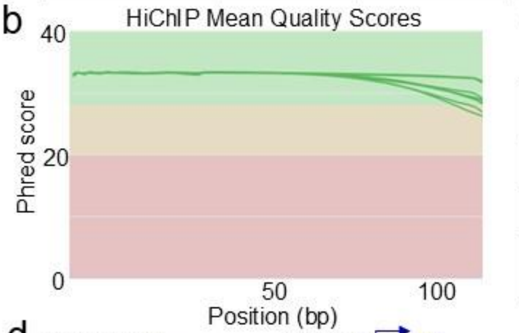


**c** HiChIP-seq percentage of valid interaction pairs

| Sample name | %Valid Pairs | %Invalid pairs) |
|---|---|---|
| Control | 86.14 | 13.86 |
| TGFB1 | 86.05 | 13.95 |
| TGFB1+*miR*-9-LOF | 87.56 | 12.44 |

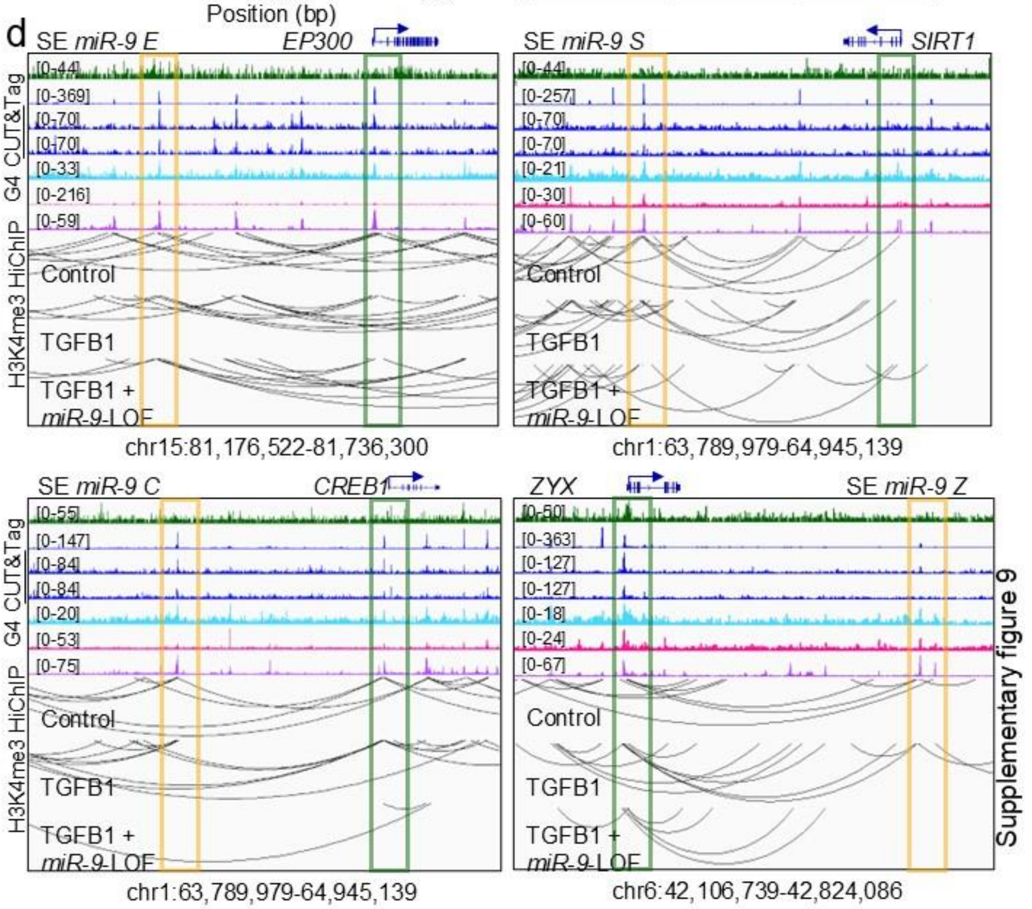


**Supplementary Figure 9: H3K4me3 HiChIP quality validation.** (**a**) Table showing the amount of reads, the trimmed, unmapped and the percentage of mapped reads. (**b**) Mean of Phred quality

score. (**c**) Table showing pair distribution into Valid Pairs and Invalid Pairs. (**d**) Visualization of promoters of selected miR-9 target genes (green squares) and SE with miR-9 enrichment (orange squares) using IGV genome browser showing enrichment miR-9 by ChIRP-seq (green), G4 by G4P ChIP-seq and G4 CUT&Tag in control and *miR-9* LOF conditions (blue), MED1 (turquoise), KLF4 (magenta) and H3K27ac (purple) by ChIP-seq. Reads were normalized using reads per kilobase per million (RPKM) measure and are represented as log2 enrichment over their corresponding inputs. Bottom, chromatin loops by HiChIP-seq in MLg cells that were transfected with control (Ctrl) or miR-9-specific antagomir (miR-9-LOF, loss-of-function), and non-treated or treated with TGFB1, as indicated. Images show the indicated loci with their genomic coordinates, the arrows show transcription direction.

| Transcript | Primer sequence (5’→3’) | |
|---|---|---|
| *Zdhhc5* | Forward | AGGTGCGAATGAAATGGTGT |
| | Reverse | TTTACCCAAGGGCAGTGATG |
| *Ncl* | Forward | TGTTGTGGATGTCAGAACTGGT |
| | Reverse | GCTCCAAGGCCTTTTCTAGG |
| *Lzts2* | Forward | GCTGTGCCACTTTTGAACG |
| | Reverse | TGAGATTCCTTCAGTTGCTGC |
| *Hdac7* | Forward | AGCTGGCTGAAGTGATCC |
| | Reverse | TCACCATCAGCCTCTGAG |
| *Prap1* | Forward | TGGGACCACTCAGCTACAAG |
| | Reverse | CCAGTTCTAAGACCTGCCC |

**Supplementary Table 1: Sequences of primer pairs used for gene expression analysis.**

| **Gene** | **Primer sequence (5'→3')** | |
|---|---|---|
| *Zdhhc5* | Forward | CCAATCACCGTTAAGCCTTT |
| | Reverse | TTTACCCCACTGACGCTTTC |
| *Ncl* | Forward | CACACCAGGAAGTCACCTCTC |
| | Reverse | TTCTTGGCTATGATGCGAGTC |
| *Lzts2* | Forward | CGAGCGCTACCGAGCACACAC |
| | Reverse | GCGCAAGTAACCCACGTCACGC |
| SE *miR-9 Z* | Forward | ACCAGGAAAGTTTGCGAGGG |
| | Reverse | CTCGAAGGAACAGACGGAGG |
| SE *miR-9 N* | Forward | AGTCCCAGTCGCCTTGTTTC |
| | Reverse | AATTCACCTGAGACTCCCGC |
| SE *miR-9 L* | Forward | TTACAGCTTTCCGACCAGGC |
| | Reverse | CCTCTCAAGCCTCTGTCCAC |

**Supplementary Table 2: Sequences of primer pairs used for analysis of gene promoter and super-enhancer regions.**
