# Supplementary material for: Nuclear microRNA 9 mediates G-quadruplex formation and 3D genome organization during TGF-β-induced transcription
Source: Nat Commun. 2024 Dec 20;15:10711. doi: 10.1038/s41467-024-54740-x (PMC11662019; doi:10.1038/s41467-024-54740-x)
Supplement: Supplementary file 2 — Description of Additional Supplementary Files [file 41467_2024_54740_MOESM2_ESM.pdf]

Description of Additional Supplementary Files

Supplementary Data 1: This is an Excel file that contains a list of all NGS data (generated by us or publicly available) that were used in the manuscript.
