# Supplementary material for: Nuclear microRNA 9 mediates G-quadruplex formation and 3D genome organization during TGF-β-induced transcription
Source: Nat Commun. 2024 Dec 20;15:10711. doi: 10.1038/s41467-024-54740-x (PMC11662019; doi:10.1038/s41467-024-54740-x)
Supplement: Supplementary file 4 — Reporting Summary [file 41467_2024_54740_MOESM4_ESM.pdf]

nature portfolio

Corresponding author(s): Guillermo Barreto

Last updated by author(s): Sep 27, 2024

Reporting Summary

Nature Portfolio wishes to improve the reproducibility of the work that we publish. This form provides structure for consistency and transparency in reporting. For further information on Nature Portfolio policies, see our Editorial Policies and the Editorial Policy Checklist.

Statistics

For all statistical analyses, confirm that the following items are present in the figure legend, table legend, main text, or Methods section.

n/a	Confirmed	
☐	☒	The exact sample size (*n*) for each experimental group/condition, given as a discrete number and unit of measurement
☐	☒	A statement on whether measurements were taken from distinct samples or whether the same sample was measured repeatedly
☐	☒	The statistical test(s) used AND whether they are one- or two-sided<br>*Only common tests should be described solely by name; describe more complex techniques in the Methods section.*
☐	☒	A description of all covariates tested
☐	☒	A description of any assumptions or corrections, such as tests of normality and adjustment for multiple comparisons
☐	☒	A full description of the statistical parameters including central tendency (e.g. means) or other basic estimates (e.g. regression coefficient) AND variation (e.g. standard deviation) or associated estimates of uncertainty (e.g. confidence intervals)
☐	☒	For null hypothesis testing, the test statistic (e.g. *F*, *t*, *r*) with confidence intervals, effect sizes, degrees of freedom and *P* value noted<br>*Give P values as exact values whenever suitable.*
☒	☐	For Bayesian analysis, information on the choice of priors and Markov chain Monte Carlo settings
☒	☐	For hierarchical and complex designs, identification of the appropriate level for tests and full reporting of outcomes
☐	☒	Estimates of effect sizes (e.g. Cohen's *d*, Pearson's *r*), indicating how they were calculated

Our web collection on statistics for biologists contains articles on many of the points above.

Software and code

Policy information about availability of computer code

Data collection	Microsoft Excel, R, GraphPrism, FastQC, MultiQC, Trimmomatic, Bowtie2, STAR, Samtools, HOMER, IGV genome browser,DAVID, KEGG, Bedtools, Deeptools, ngsplot, ROSE, DEseq2, R studio, ImageJ, ImarisViewer, ChIPseeker, MACS2, MEGA11, MEME-ChIP.
Data analysis	Microsoft Excel, R-scripts and GraphPad Prism were used to analyze the statistics. All the plots were made using R studio, GraphPad Prism, deeptools, ngsplot, and Microsoft Excel. Sequencing raw reads were visualized by FastQC and MultiQC. Low quality reads were filtered out by using trimmomatic. Bowtie2 or STAR were used for mapping of trimmed sequencing reads. The sam files were converted to bam format by using samtools. HOMER was used for making Tag libraries, quantification, annotation, analysis for HiChIP data including finding hubs and interactions. NGS data was visualized using IGV genome browser including HiChIP interactions. DAVID and KEGG were used to perform Gene Set Enrichment Analysis (GSEA). Typical- and Super-enhancers were assessed using ROSE. Differential expressed genes were analysed using DEseq2. Image J was used for densiometric analyses and fluorescence microscopy image processing. ImarisViewer was used to process 3D confocal images. ChIPseeker was used to generate chromosomal distribution coverage plot. MACS2 was used for peak calling of NGS data. MEGA11 was used for phylogeny analysis and representation. Motif enrichment analysis was done using MEME-ChIP.

For manuscripts utilizing custom algorithms or software that are central to the research but not yet described in published literature, software must be made available to editors and reviewers. We strongly encourage code deposition in a community repository (e.g. GitHub). See the Nature Portfolio guidelines for submitting code & software for further information.

Data

Policy information about availability of data

All manuscripts must include a data availability statement. This statement should provide the following information, where applicable:

- Accession codes, unique identifiers, or web links for publicly available datasets
- A description of any restrictions on data availability
- For clinical datasets or third party data, please ensure that the statement adheres to our policy

All the sequencing data were submitted to Gene expression Omnibus (GEO) under the accession number GSE244952. The mass spectrometric raw data to the ProteomeXchange Consortium with dataset identifier: PXD054375, via the MassIVE partner repository (https://massive.ucsd.edu/, MassIVE-ID: MSV000095480; doi:10.25345/C5639KH31:).

Research involving human participants, their data, or biological material

Policy information about studies with human participants or human data. See also policy information about sex, gender (identity/presentation), and sexual orientation and race, ethnicity and racism.

Reporting on sex and gender	NA
Reporting on race, ethnicity, or other socially relevant groupings	NA
Population characteristics	NA
Recruitment	NA
Ethics oversight	NA

Note that full information on the approval of the study protocol must also be provided in the manuscript.

Field-specific reporting

Please select the one below that is the best fit for your research. If you are not sure, read the appropriate sections before making your selection.

[x] Life sciences   [ ] Behavioural & social sciences   [ ] Ecological, evolutionary & environmental sciences

For a reference copy of the document with all sections, see nature.com/documents/nr-reporting-summary-flat.pdf

Life sciences study design

All studies must disclose on these points even when the disclosure is negative.

Sample size	No statistical method was used to pre-select the sample size. We performed each experiment 3 times independent from each other, unless it is indicated differently. In each experiment the samples were measured at least in duplicate. Depending on the data, different tests were performed to determine the statistical significance of the results. ChIRP-seq, CUT&Tag, RNA-seq, ChIP-seq, HiChIP was analyzed by deep sequencing using a single sample and confirmed by single gene analysis. The values of the statistical tests used in the different experiments can be found in the Source data file.
Data exclusions	No data were excluded from the analyses.
Replication	For each experiment, all attempts at replication were successful.
Randomization	No method of randomization was used. Experiments were performed 3 times independent from each other, unless it is indicated differently. In each experiment the equivalent samples were treated as equally as possible. Statistical relevant results were reproducible.
Blinding	NA

Reporting for specific materials, systems and methods

We require information from authors about some types of materials, experimental systems and methods used in many studies. Here, indicate whether each material, system or method listed is relevant to your study. If you are not sure if a list item applies to your research, read the appropriate section before selecting a response.

Materials & experimental systems

n/a	Involved in the study
[ ]	[x] Antibodies
[ ]	[x] Eukaryotic cell lines
[x]	[ ] Palaeontology and archaeology
[x]	[ ] Animals and other organisms
[x]	[ ] Clinical data
[x]	[ ] Dual use research of concern
[x]	[ ] Plants

Methods

n/a	Involved in the study
[ ]	[x] ChIP-seq
[x]	[ ] Flow cytometry
[x]	[ ] MRI-based neuroimaging

Antibodies

Antibodies used	Reported in Material and Methods section. Primary antibodies used in this study are FITC anti-Biotin (Abcam, # ab53469), H3K4me3 (Abcam, # ab8580). Alexa 488 (Invitrogen, # A11008) Alexa 594 (Invitrogen, # A11005) BG4 (Millipore, # MABE917) anti-IgG (Santa Cruz, # sc-2027) H3K27ac (Abcam, # ab4729). mouse anti-FLAG antibody (Sigma, # F1804) rabbit anti-mouse (Sigma, # M7023) guinea pig anti-rabbit antibody (Novus Biologicals, # NBP1-72763) SMARCA5 (Invitrogen, # MA5-35378), LMNB1 (Santa Cruz, # sc-374015), LMNA (Santa Cruz, sc-20681), CHD4 (Abcam, # 70469), RAD21 (Abcam, # ab992), GAPDH (Sigma, # MFCD01322099)
Validation	Species validation of all primary antibodies used in this study can be found in the corresponding manufacturer's websites.

Eukaryotic cell lines

Policy information about cell lines and Sex and Gender in Research

Cell line source(s)	MLg (ATCC CCL-206), MLE-12 (ATCC CRL-2110), NMuMG (ATCC CRL-1636), human primary lung fibroblasts, MFML4
Authentication	ATCC validates their cell lines.<br>Primary human fibroblasts were obtained from UGMLC Giessen Biobank and validated by parent labs and Rubio et al.2019, Nature Communications.<br>MFML4 was characterized and validated by Akeson et al.2000, Developmental dynamics : an official publication of the American Association of Anatomists.
Mycoplasma contamination	All cell lines used in this study were tested negative of mycoplasma contamination.
Commonly misidentified lines (See ICLAC register)	NA

Plants

Seed stocks	NA
Novel plant genotypes	NA
Authentication	NA

ChIP-seq

Data deposition

- [x] Confirm that both raw and final processed data have been deposited in a public database such as GEO.
- [x] Confirm that you have deposited or provided access to graph files (e.g. BED files) for the called peaks.

Data access links<br>*May remain private before publication.*	GSE244952<br>Reviewer password= mxqrmqwufdwxpmr
Files in database submission	GSM7832497 01MLg_Mir9_ChIRP<br>GSM7832498 02MLg_Inp_ChIRP<br>GSM7832499 01MLg_K4m3_HICHIP_Ctr<br>GSM7832500 02MLg_K4m3_HICHIP_Tgfb

GSM7832501 03MLg_K4m3_HICHIP_Tgfb_antiMir9<br>GSM7832502 04MLg_K4m3_HICHIP_Mir9_LOF<br>GSM7832503 01MLg_RNAs_riZero_Ctr<br>GSM7832504 02MLg_RNAs_riZero_Mir9_LOF<br>GSM7832505 03MLg_RNAs_riZero_Tgfb<br>GSM7832506 04MLg_RNAs_riZero_Tgfb_Mir9_LOF<br>GSM8440910 MLg_H3K4me3_CaT_Ctr<br>GSM8440911 MLg_H3K4me3_CaT_Mir9-LOF<br>GSM8440912 MLg_H3K27ac_CaT_Ctr<br>GSM8440913 MLg_H3K27ac_CaT_Mir9-LOF<br>GSM8440914 MLE-12_H3K4me3_CaT_Ctr<br>GSM8440915 MLE-12_H3K4me3_CaT_Mir9-LOF<br>GSM8440916 MLE-12_H3K27ac_CaT_Ctr<br>GSM8440917 MLE-12_H3K27ac_CaT_Mir9-LOF<br>GSM8440918 MLg_Ctr_G4_CaT_r1<br>GSM8440919 MLg_Mir9LOF_G4_CaT_r1<br>GSM8440920 MLE-12_Ctr_G4_CaT_r1<br>GSM8440921 MLE-12_Mir9LOF_G4_CaT<br>GSM8440922 x01Mlg_Ctr_H3K4me3_ChIP<br>GSM8440923 x02Mlg_TGFB_H3K4me3_ChIP<br>GSM8440924 x03Mlg_TGFB_Mir9LOF_H3K4me3_ChIP

Genome browser session (e.g. UCSC)	NA

Methodology

Replicates	One sample was sent for sequencing experiments
Sequencing depth	Quality measures for all the sequencing experiments can be found in the supplementary figures S2a, S3a, S3c, S4a, S5b, S6a, S8a, S9a, S9b, S9c
Antibodies	Reported in Material and Methods section. Primary antibodies used in this study are H3K4me3 (Abcam, # ab8580), BG4 (Millipore, # MABE917) anti-IgG (Santa Cruz, # sc-2027) H3K27ac (Abcam, # ab4729), mouse anti-FLAG antibody (Sigma, # F1804) rabbit anti-mouse (Sigma, # M7023) guinea pig anti-rabbit antibody (Novus Biologicals, # NBP1-72763)
Peak calling parameters	Peaks calling for H3k4me3 marker MACS3 callpeak, with settings (--broad -g mmu -q 0.001 --keep-dup 1 --fix-bimodal --nomodel --extsize 1000). Peaks for H3k27ac were called with MACS3 callpeak, settings (--broad -g mmu -q 0.01 --keep-dup 1 --fix-bimodal --nomodel --extsize 200) and G4 was called with MCAS14 setting (p1e3 for the p-value).<br>To calculate the broadness of peaks, we first performed summary statistics of the peak size from the merged peak list. If the size of a peak was equal to or higher than the top 75 % of peaks quantile 3 (Q3) was consider wide (≥ 2.7kb). If the peak was between Q3 and Q2 was consider medium size peak (≥ 2 kb and < 2.7 kb). The narrow peaks were in the bottom 25% peaks, Q1 or less (< 2kb).
Data quality	Raw reads were visualized using FastQC or MultiQC. Low quality reads were discarded using trimmomatic v0.32. Trimmed reads were further used for processing.
Software	Bowtie2 or STAR were used for mapping of trimmed sequencing reads. The sam files were converted to bam format by using samtools. HOMER was used for making Tag libraries, quantification, annotation, analysis for HiChIP data including finding hubs and interactions. NGS data was visualized using IGV genome browser including HiChIP interactions.
